# Could be FOXO3a, miR-96-5p and miR-182-5p useful for Brazilian women with luminal A and triple negative breast cancers prognosis and target therapy?

**DOI:** 10.1016/j.clinsp.2022.100155

**Published:** 2023-01-19

**Authors:** Daniele Carvalho Calvano Mendes, Carlos Marino Cabral Calvano Filho, Natália Garcia, Marcos Desidério Ricci, José Maria Soares, Katia Candido Carvalho, Edmund Chada Baracat

**Affiliations:** a Laboratório de Ginecologia Estrutural e Molecular (LIM 58), Disciplina de Ginecologia, Departamento de Obstetrícia e Ginecologia, Hospital das Clínicas da Faculdade de Medicina da Universidade de São Paulo, HCFMUSP, São Paulo, SP, Brazil; b Setor de Mastologia, Disciplina de Ginecologia, Universidade de Brasília, Brasília, DF, Brazil

**Keywords:** Breast cancer, miRNAs, FOXO3a, qRT-PCR, IHC

## Abstract

FOXO3a dysregulation is frequently implicated in tumorigenesis, and its inhibition can occur by several molecular mechanisms. Among these, post-transcriptional suppression by miRNAs has been associated with various cancers initiation. Here, we assessed the expression profiles of the most relevant miRNAs for breast tumorigenesis, using Luminal A (LA) and Triple-Negative (TN) breast cancer from Brazilian patients, by the quantitative real time-PCR method. Their potential prognostic role for the patients was also evaluated. We identified the miRNAs *miR-96-5p* and *miR-182-5p*, de-scribed as negative regulators of FOXO3A, with differential expression both in LA and TN tumors when compared to normal tissue. The *miR-96-5p* and *miR-182-5p* miRNAs were upregulated in LA (7.82 times, p < 0.005; 6.12 times, p < 0.005, respectively) and TN breast cancer samples (9.42 times, p < 0.0001; 8.51 times, p < 0.0001) compared to normal tissues. The samples with higher *miR-96-5p* and *miR-182-5p* expression (FR ≥ 4) were submitted for FOXO3a immunostaining. Reduced protein detection was observed in all of the tumors compared to normal tissues. The most prominent miRNA expression and FOXO3a protein suppression were observed in TN samples (p < 0.001), indicating the relevant role of these molecules in this tumor biology and clinical behavior. Our results corroborate the literature regarding to the relevance of FOXO3a in the breast cancer, and they open new perspectives for alternative target therapy options for Brazilian patients expressing both FOXO3a and its regulatory miRNAs.

## Highlights

• *miR-96-5p* and *182-5p* are associated with FOXO3a dysregulation and cancer.
• FOXO3a and miRNAs represent molecular markers in Brazilian breast cancer patients.

## Introduction

Breast cancer is the most frequent malignant tumor among women, with the exception of non-melanoma skin cancers. It corresponds to 24.2% of all female malignant tumors, and its incidence rate is 46.3/100,000.^1^ In Brazil in 2018, there were 17,572 deaths due to breast cancer in women, which corresponds to 16.4% of cancer deaths in the female population.^2^ Though it is considered to have a relatively good prognosis, breast cancer mortality rates remain high in the country. The mean five-year survival in developed countries has increased slightly, reaching 85%. However, in developing countries, the survival is approximately 60%.^3^

The main landmarks of mammary carcinogenesis are related to proliferation, differentiation, and apoptosis. The molecular interactions involved in these processes are not fully understood yet. In fact, the network of mechanistic and physical interactions among genes, proteins, and metabolites remains largely unexplored due to the immense complexity of the connections among the various pathways regulating these cellular processes.^4^ Studies that have applied theoretical approaches to explore genes directly involved in the pathogenesis of breast cancer have revealed a network of similarities, including the ERBB, PI3K-AKT, mTOR, FOXO, p53, HIF-1, VEGF, MAPK, and prolactin-signaling pathways.^4^^,^^5^

The classification of breast cancer with the traditional parameters has been supplemented with categorizations based on its gene expression.^6^, ^7^, ^8^ The most popular expression-typing platforms include MammaPrint®, Oncotype DX®, the two-gene model, the genomic grade index, and the intrinsic subtype model. The latter includes broad biological information and allows the classification of tumors into the subtypes luminal A, luminal B, normal-like, Human Epidermal growth factor Rreceptor 2 (HER-2) / ErbB2-positive, and basaloid.^7^, ^8^, ^9^

Luminal A (LA) tumors are a well-differentiated type of breast cancer that co-express sexual hormone receptors, and the most prevalent type with low rate of regional or distant recurrence.^1^^,^^10^ In addition, LAs have a more favorable natural biology and clinical progression, as well as a well-established targeted therapy. Their recurrences are late and generally associated with resistance to endocrine therapy development.^7^, ^8^, ^9^, ^10^ On the other hand, patients with the Triple-Negative (TN) subtype are younger at the time of diagnosis and have larger, undifferentiated, and highly proliferative tumors without sexual receptors expression and frequent visceral metastases.^9^ TNs represent 15%–20% of all breast cancers and they are usually an invasive ductal cancer. Approximately 80% are classified as basal-like. They are tumors with a very aggressive phenotype and various recurrence patterns.^11^ They present in more advanced stages and are associated with a lower 5-year survival rate (77% vs. 93%) than the non-triple-negative phenotypes.^12^ Their prognosis is worse than luminal A, indicating that the regulatory mechanisms may be different between TN and LA.

It is known that each tumor has its own molecular identity and, nowadays, several researchers seek to elucidate the mechanisms involved in the different breast cancer phenotypes arising. Studies have addressed topics such as DNA methylation, histone acetylation, RNA interference, and gene regulation by miRNAs.^13^^,^^14^ Studies focusing on the epigenetic mechanisms have shown the involvement of miRNAs in multiple biological functions, as regulators of gene expression, cell proliferation, apoptosis, metabolism, angiogenesis, and others.^15^ The differential expression of miRNAs due to chromosomal alterations can be responsible for both cancer development and progression.^16^ Furthermore, their expression patterns differ for specific tissues and differentiation states.^17^

The molecular mechanisms of miRNAs and their roles in the physiological and pathological processes, as well as their correlation with prognosis and treatment prediction in patients with breast cancer are still poorly understood. However, it is well-established in the literature that miRNAs may control tumor metastasis and treatment resistance through the regulation of divergent or convergent signaling pathways.^18^ In this sense, FOXO3a signaling is widely implicated in a variety of cancers, including breast cancer,^19^ and its 3`-Untranslated Region (3’-UTR) harbors several miRNA target sequences.^20^ FOXO3a dysregulation has been reported in breast cancer,^21^^,^^22^ as it acts in a variety of cellular processes, including apoptosis, proliferation, cell cycle progression, DNA damage, and tumorigenesis.

Here, we tried to identify biomarkers based on the miRNA expression profiles from LA and TN breast cancer in a set of Brazilian samples. The choice for these two tumor types was mainly based on their very different clinical and biological behavior, focusing on patients’ prognosis and treatment perspectives. We assessed the expression profile of 84 oncomir sequences, evaluating their expression profiles and their correlation with the clinical pathological features of the patients included in our sample set. Additionally, the expression of the FOXO3a protein was also assessed as a relevant target gene for miRNA regulation in breast cancers.

## Materials and methods

### Samples

This is a descriptive study of miRNA expression profiles in Luminal A (LA) and Triple-Negative (TN) breast cancer, using Formalin-Fixed and Paraffin-Embedded (FFPE) samples. The tumors were obtained from surgical resections of selected patients with invasive ductal carcinoma who were treated and followed up at the Setor de Mastologia, Disciplina de Ginecologia, the Hospital das Clínicas, Faculdade de Medicina, Universidade de Sao Paulo, and the Instituto do Cancer do Estado de Sao Paulo (ICESP). The study was approved by the institutional review board CAPpesq (0042/11).

The FFPE samples included (1) 33 LA tumors with the following characteristics: invasive breast ductal carcinoma, ER- and PR-positive and HER-2 negative with Ki67 < 15%; (2) 31 TN tumors with the following characteristics: invasive breast ductal carcinoma, ER- and PR-negative and HER-2 negative. Normal breast tissues from the same patients (matched normal tissues) were used as control (reference) samples.

### qRT-PCR for the detection of mirna expression profiles

The RNA isolation from the FFPE tissue was performed using the miRNeasy FFPE® Kit (Qiagen Sciences, Frederick, MD, USA) following the manufacturer´s instructions. cDNA synthesis was carried out using the miScript II RT® Kit (Qiagen Sciences, Frederick, MD, USA), exclusively using the RNA samples that showed the necessary quality for PCR assays. The profiles of the gene expressions of miRNA were evaluated using the Human Breast Cancer miScript miRNA PCR Array® (code number MIHS 109Z, Qiagen Sciences, Frederick, MD, USA), including 84 microRNA sequences potentially related to the diagnosis, staging, and prognosis of breast cancer. To ensure the quality control of the reactions, the plate also included two miRNA extraction controls, six housekeeping snRNAs, two reverse transcription reaction controls, and two positive PCR controls. The quantitative Real-Time PCR (qRT-PCR) reactions were carried out in an ABI 7500 Real-Time PCR System (Applied Biosystems). The quantitation of the gene expression was assessed using the ΔΔCT relative quantification method, as previously described.^23^ This was used as a cut-off value for the fold regulations of ≤-4 and ≥4 to determine the up- and downregulation of the miRNAs.^23^

In the end, 57 samples passed for the plate quality control, which included cDNA efficiency control, genomic DNA contamination, PCR amplification efficiency, and housekeeping gene amplification profiles. The initial gene expression analyses included 13 normal samples, 20 LA samples, and 24 TN samples, and the results are presented as the Fold Regulation (FR), which indicates the times that a gene is more or less expressed in the tumors compared to the normal tissues.

### Tissue microarray (TMA) slides and Immunohistochemistry (IHC) Reactions

For FOXO3a protein analyses, only the samples that presented upregulation (FR ≥4) of *miR96-5p* and *miR-182-5p* expression, both negative regulators of FOXO3a, were selected. Our goal was to observe whether there was a significant correlation between the miRNAs and FOXO3a protein expressions in these samples. In total, 33 samples were included (8 LA, 18 TN, and 7 normal breast samples) in the TMA block.

The FOXO3a protein was selected as a potential target based on the expression of the miRNAs identified by qRT-PCR. The IHC staining was performed as previously described.^24^ Briefly, the antigens were retrieved using sodium citrate buffer with a pH of 6.0 for 15 minutes in a pressure cooker (Pascal, Dako, Denmark). All IHC reactions were standardized on conventional slides before the final analysis was performed on the TMA slides. As the primary antibody, we added the polyclonal antibody of FOXO3a (1:100, rabbit, Novus Biological Inc, USA), with overnight incubation at 4°C. As a positive control, the tissue indicated by the manufacturer was included. All IHC reactions were performed in duplicate.

Two independent pathologists performed the semi-quantitative analysis of the tissue staining as a double-blind analysis, and all discordant cases were retrieved for discussion and consensus. The final score of the protein expression was obtained using the intensity of the staining (0, no staining; 1, weak; 2, mild; 3, strong staining), and the frequency of stained cells (0, negative; 1, ˂ 10% of the cells; 2, 10%–50% of the cells; 3, 50%–75% of the cells; 4, ˃ 75% of the cells). The multiplication of these two scores (0 to 12) by sample was used for statistical analyses and graphic representation. The expression of FOXO3a protein was considered to be positive when the average staining score was ≥ 3. For statistical analyses, we grouped the expression data as follows: negative/weak (≤ 3), moderate (> 3 and ≤ 8), or strong (> 8 and ≤ 12).^24^ Negative controls were obtained by omitting the primary antibody or including nonreactive IgG.

### Statistical analysis

All statistical analyses were performed using GraphPad Prism 5.0 (GraphPad Software, San Diego, CA, USA) and SPSS version 21 (IBM Corp., Armonk, NY, USA) for Windows. The expression levels of the miRNAs in the LA and TN groups were analyzed with the Kruskal-Wallis test. Dunn's multiple comparison post-test was applied to compare the differences among the miRNAs in the groups. When we compared the miRNA or FOXO3a expression between the two groups (LA or TN vs. normal tissues), we used the Mann-Whitney U non-parametric test. For the comparison of the three groups, we used ANOVA.

The Pearson and Spearman's rank correlation coefficient was used to measure the strength of the linear relationship between two random variables for the parametric and non-parametric data, respectively. A Chi-Square test and Fisher's exact test were used to analyze the differences among the frequencies of each variable. The patients’ ages are represented by the mean and standard deviation (mean ± SD). Differences are considered statistically significant at p < 0.05.

## Results

**Fig. 1:**
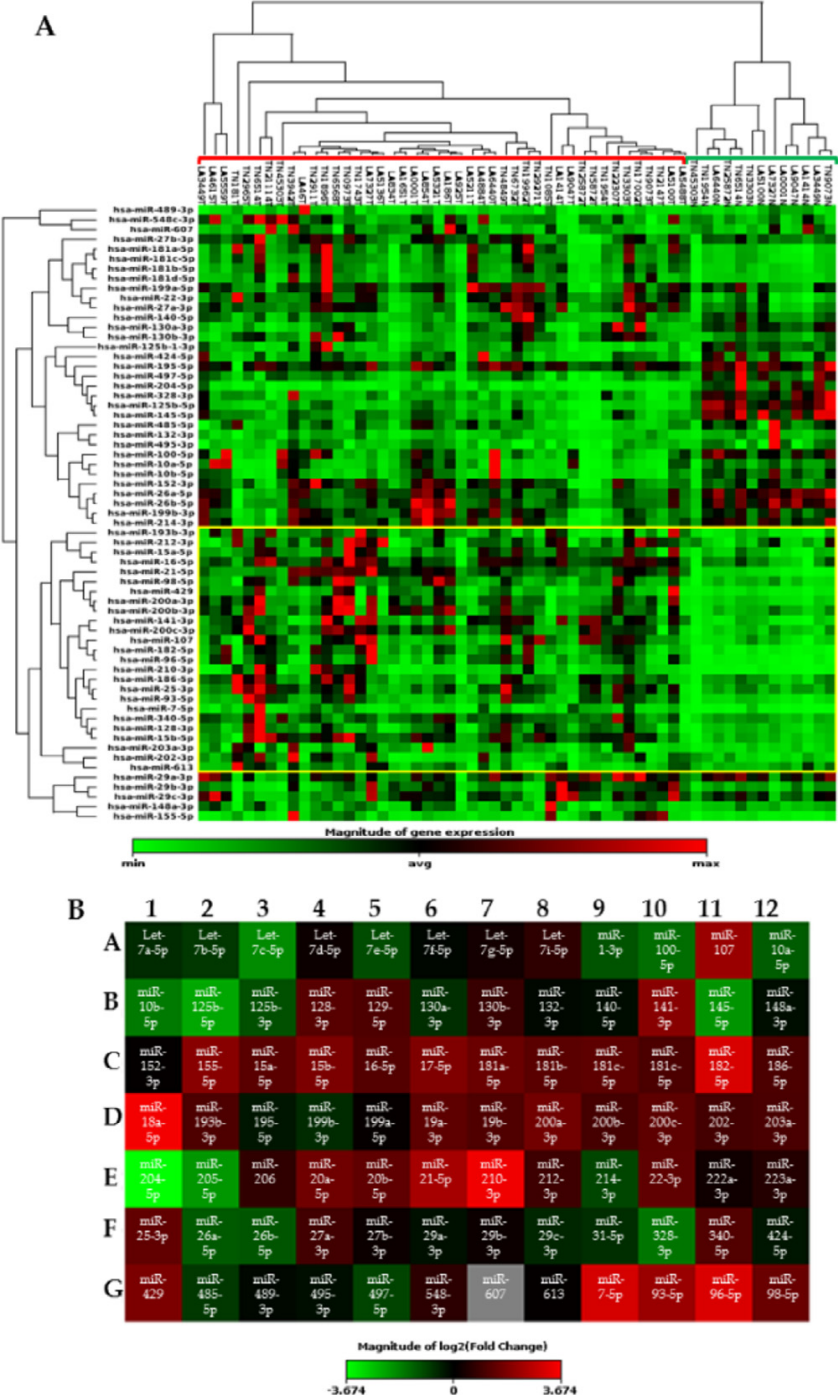
(A) Clustergram showing the global gene expression profile of the miRNAs evaluated by the qRT-PCR method using all 57 samples (including 13 normal, 20 LA, 24 TN). The sample names, as well as the miRNA identifications, are indicated in the figure borders. The green and red lines at the top of the figure separate the normal from the tumor samples. The yellow square indicates the region where we found a clear separation between the tumors and normal samples considering the expression profiles of the miRNAs. The region without gene expression was excluded from the figure. (B) Heatmap showing all the miRNA sequences included in the array and their expression profile considering the tumors vs. normal tissue. The red color indicates overexpression, while the green one indicates downregulated genes. No differences between the tumor samples and normal tissues are indicated by the black color. The color scale bar indicates the grade of significance according to the gene expression values (from -5 to 5) compared to the reference (normal tissues). Yellow square indicates the region that separated the samples in two major clades, based on the miRNAs expression amount (including miR-96-5p and miR-182-5p), even without normalization.

All the FFPE selected samples were processed for RNA extraction (67 tumors and their corresponding normal tissues). Only cases with genetic material enough were submitted to cDNA syntheses. In the end, only 57 samples were included in the array analyses indeed. Some samples were lost due to several steps of quality controls necessary to avoid bias in the gene expression results. In the end, 13 normal samples, 20 LA samples, and 24 TN samples were evaluated by qRT-PCR (Fig. 1). We found several sequences with differential expression, comparing the LA and TN samples with the normal tissues. The clustergram shows the gene expression profile observed for each sample compared to the reference group (normal samples) using a color scale. This analysis provides no normalized or hierarchized results that allow us to observe the general expression profile of the genes individually for each sample. Even with this preliminary data, we can observe a region in the figure with a clear separation between tumors and normal tissues considering the expression values of the miRNAs (yellow square, Fig. 1). Regions without gene variations were removed from the figure. Figure 1B shows all the miRNAs included in the array and the expression profile for each one considering the expression values of the tumors grouped together compared to normal samples, using the same color scale described in Figure 1A.

**Fig. 2:**
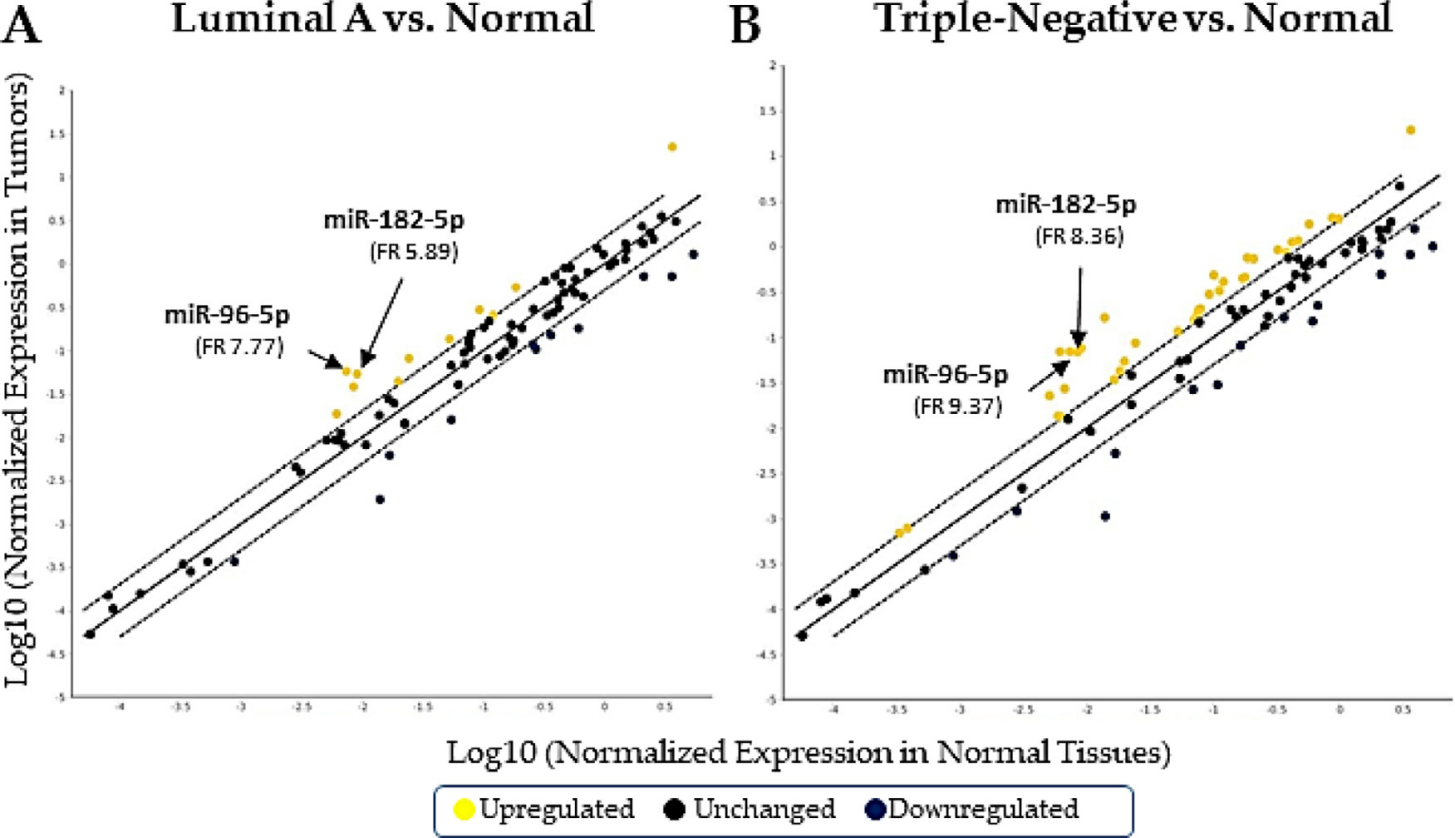
Expression profile of the 84 miRNA sequences included in the array analyses. After data normalization, 11 miRNAs were found upregulated (yellow circles) in the luminal A samples and another 11 were downregulated (blue circles). Comparing the triple-negative and normal tissues, 32 miRNAs were upregulated and 15 were downregulated. The black circles indicate no difference between the tumors and normal tissues. The positions and fold regulations of miR-96-5p and miR-182-5p (FR) are indicated in the graphs.

The supervised categorization was performed according to the magnitude of the expression of the 84 miRNAs (Fig. 2). After data normalization, we found 11 miRNAs were upregulated and another 11 were downregulated in the LA samples (Fig. 2A), using normal tissues as reference. For the TN samples, a high number of molecules were identified compared to the reference samples; 32 miRNAs were upregulated and 15 were downregulated (Fig. 2B). Among all the differentially regulated miRNAs, we found that miR-96-5p and miR-182-5p were upregulated (5.89 to 9.37 times) in the tumors (Fig. 2 A and B). Both *miR-96-5p* and *182-5p* were previously associated with breast cancer initiation and prognosis, and they share an important potential tumor suppressor gene ‒ FOXO3a ‒ as a target.

**Fig. 3:**
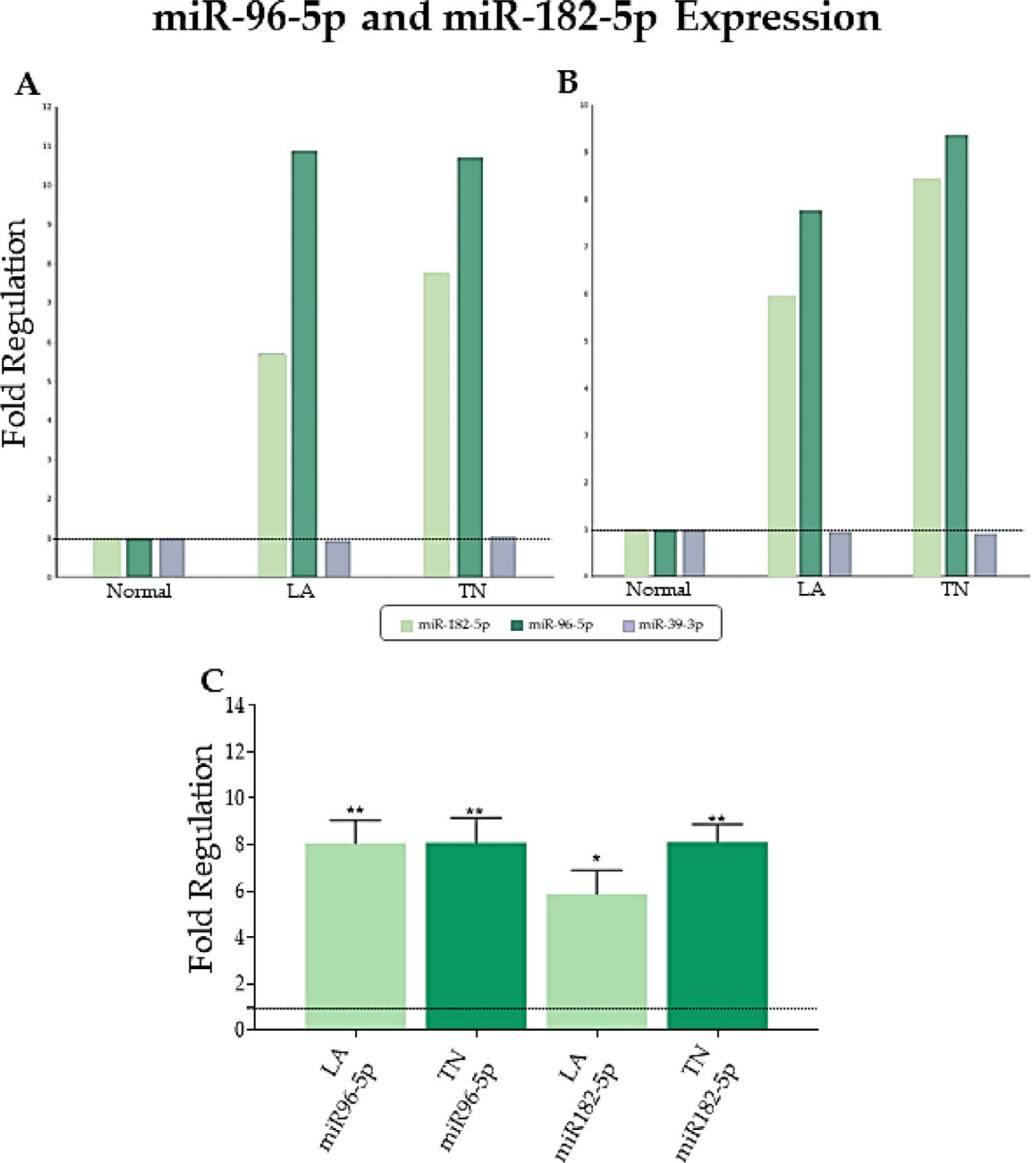
Gene expression profile of miR-96-5p and miR-182-5p in the samples. (A) SA Biosciences software date for the initial analyses including all 57 samples. (B) SA Biosciences software data including the samples selected as upregulates for both miR-96-5p and miR-182-5p (n = 33). miR-39-3p was selected as a fold regulation reference value, but the geometric mean of the 4 endogenous controls was used for data analysis. (C) Values of expression obtained using the DDCT method for individual samples (n = 33), using normal tissues as a reference for gene expression. LA, Luminal A group or samples; TN, Triple-Negative group or samples; *p < 0.005, **p < 0.0001.

The literature shows the involvement of FOXO3a in the pathogenesis of breast cancer.^19^^,^^21^^,^^22^ Thus, based on studies showing the interaction of *miR-96-5p* and *miR-182-5p* with FOXO3a regulation, we decided to explore the correlation between the expression levels of *miR-96-5p* and *miR-182-5p* with the expression levels of the FOXO3a protein in luminal A and triple-negative breast cancer compared to normal breast tissue. For that, we selected only the samples with an FR ≥4 for *miR-96-5p* and *182-5p* expression values, resulting in 33 samples (8 LA, 18 TN, and 7 normal breast samples) to continue the analyses (Fig. 3). Only for statistical purposes, we exported the expression data from the SA Biosciences software and carried out the DDCT analysis manually. Figure 3 shows the *miR-96-5p* and *miR-182-5p* expression values (FR) initially considering all 57 samples (Fig. 3A), and later, only from the samples with an FR ≥ 4 (Figs. 3 B and C). Compared to normal samples, the software found *miR-96-5p* with an FR = 7.82 and 9.42, while *miR-182-5p* showed an FR = 6.12 and 8.51, respectively, in the LA and TN (Figure 3B).

Manual DDCT analyses showed *miR-182* with an FR = 5.9 and 8.1, while *miR-96-5p* showed an FR = 8.0 and 8.1 in LA and TN samples, respectively (Fig. 3C). Statistical analyses showed significant differences for the two miRNAs in the tumor samples compared to normal tissues.

**Fig. 4:**
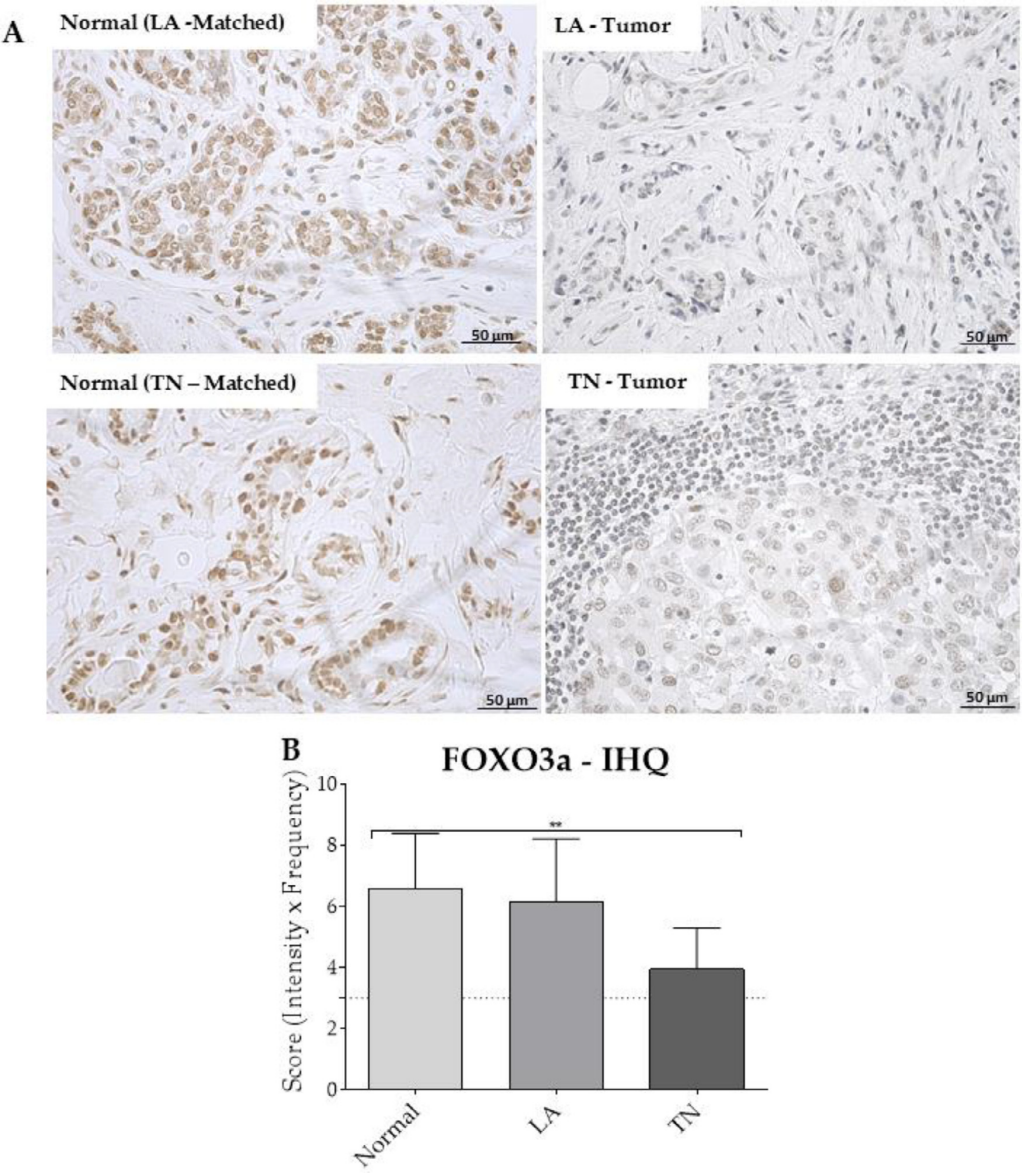
FOXO3a protein expression profiles of the samples (n = 33). (A) Photomicrographs representative of the protein nuclear staining in the patient samples. Normal tissue samples matched with Luminal A (LA-matched) and Triple-Negative (TN-matched). (B) Semi-quantitative analyses of the protein expression using the intensity and frequency of immunostaining. Only samples with miR-96-5p and miR-182-5p showing FR values ≥ 4 were included in the analyses. The dotted line indicates the cutoff value for positive reactions (> 3). **p < 0.0001 was obtained comparing TN vs. normal tissues. LA, Luminal A; TN, Triple-Negative, IHQ, Immunohistochemistry.

To validate the effect of the *miR-96-5p* and *miR-182-5p* in the expression of FOXO3a, IHC analyses were performed in the LA, TN, and matched normal breast samples that had upregulation of the two miRNAs (Fig. 4). The FOXO3a functional protein is expressed in the nuclear compartment of the cells, but, when phosphorylation occurs, the protein can be found in the cytoplasm, where it is degraded. Here, we focused on the nuclear lack of this protein expression, but the slide assessment did not show relevant cytoplasmic staining of FOXO3a. The IHC pattern of FOXO3a detection in the normal and tumor samples can be seen in Figure 3A. Semi-quantitative analyses of FOXO3a protein detection did not show significant differences between the LA and TN breast tumors, or between the LA and normal tissues. However, a significant lack of this protein expression was observed in the TN samples compared to normal samples (Fig. 3B).

**Fig. 5:**
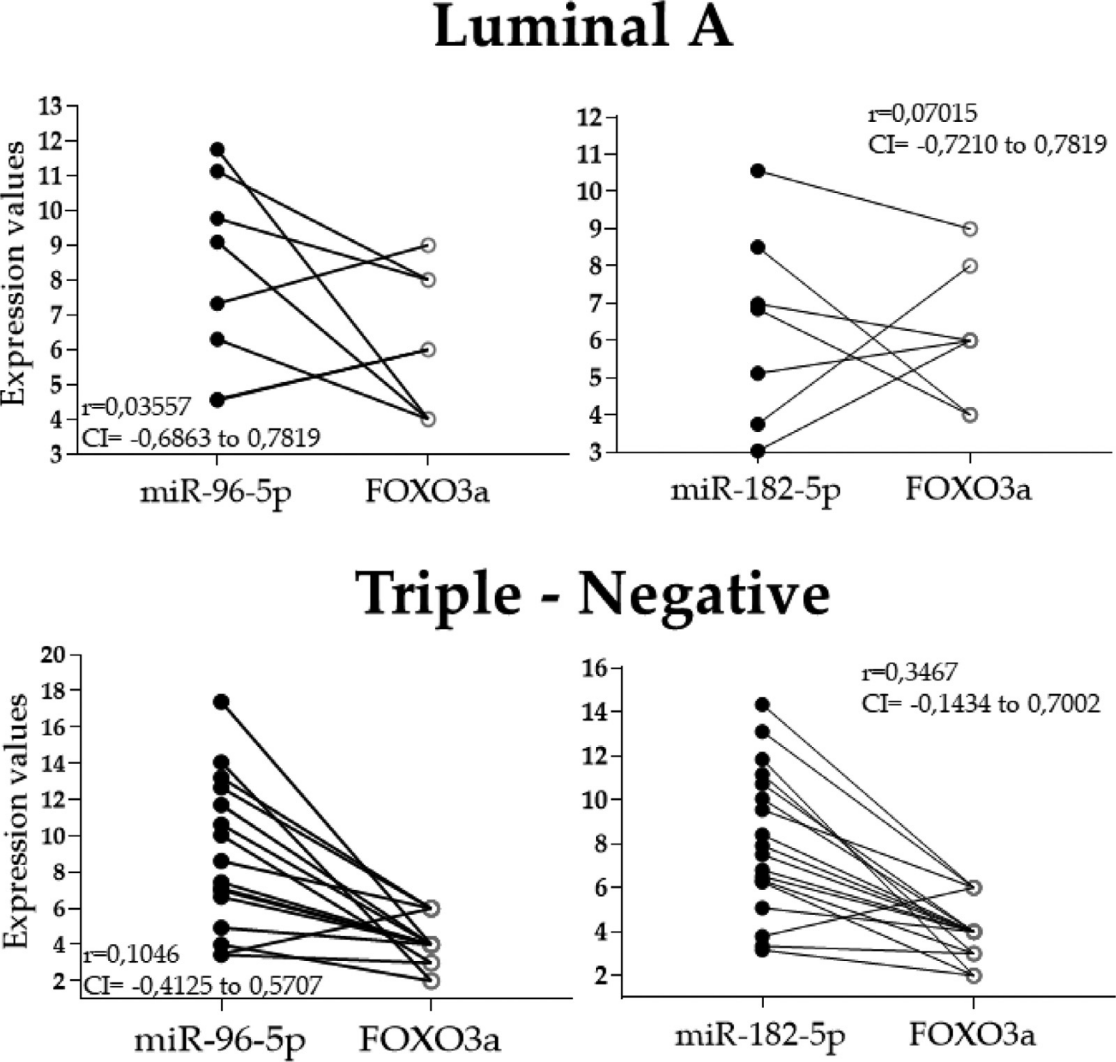
Graphs showing the relationship between the FOXO3a protein and the two miRNA (96-5p and 182-5p) expression profiles in the luminal A (n = 8) and triple-negative (n = 18) samples. Although no significant correlation was observed, a few samples did not present an inverse expression profile between the miRNAs and FOXO3a protein. The Spearman correlation test values (*r*), as well as the Confidence Interval (CI), are indicated. Some samples are overlapping because of their expression values.

Spearman's analyses did not find a significant correlation between the miRNAs and the protein expression data in the tumors, probably due to the homogeneity among the samples for all variables or the sample size. However, the inverse relationship between the miRNAs *96-5p* and *182-5p* and FOXO3a can be observed in Figure 5. Only a few samples did not present protein expression values inversely proportional to that of the miRNAs.

The clinical and pathological patients’ features were recovered to evaluate whether the miRNAs or FOXO3a expression could be relevant to the prognosis or treatment response for LA and TN breast cancer. The data collected from LA patients show that no case of perineural invasion occurred; only one patient presented recurrence and familiar history of ovarian cancer, one patient had lymphatic invasion and no “in situ” component, and all of them were > 50 years old and had children. The other information about the LA patients is presented in Table 1.

**Table 1:** Clinical and anatomopathological features of the luminal A patients (n = 6).

Variables	Categories	n (%)
Age	> 50 years	8 (100)
	≤ 50 years	0
Breastfeeding	Yes	3 (37.5)
	No	‒
	NA	5 (65.5)
Menopause	Yes	7 (87.5)
	No	1 (12.5)
Treatment	QT	1 (12.5)
	RxT	3 (37.5)
	QT + RxT.	2 (25)
	No	2 (25)
Surgery type	Mastectomy	3 (37.5)
	Quadrantectomy	5 (62.5)
Staging^a^	1	4 (50)
	2	4 (50)
Tumor size^b^	1	4 (50)
	2	4 (50)
	3	0
Histological grade^c^	1	6 (75)
	2	2 (25)
Nuclear grade^d^	1	0
	2	8 (100)
Hormonal status	ER	60%–90%
	PR	0%–70%
Ki 67	≤10%	1 (12.5)
	> 10%	7 (87.5)
Inflammatory infiltrate	Yes	8 (100)
	No	0
Death	Yes	0
	No	8 (100)

a 1 = I/IIA, 2 = IIB+

b 1 ≤ 2 cm, 2 ≥ 2.1–5, 3 ≥ 5 cm

c 1 = I+II, 2 = III

d 1 = low + intermediary, 2 = high.

For the TN breast cancer patients, 67% of the patients were > 50 years old, only one was EGFR positive and presented lymphatic invasion. The rest of the information about these women is presented in Table 2.

**Table 2:** Clinical and anatomopathological features of the TNBC patients (n = 18)^a^.

Variables	Categories	n (%)
Age	> 50 years	12 (67)
	≤ 50 years	4 (22)
Breastfeeding	Yes	13 (72)
	No	1 (5.5)
Menopause	Yes	11 (62)
	No	5 (28)
Hormone Replacement Therapy (HRT)	Yes	2 (11)
	No	14 (78)
	NA	
Familiar history (Cancer)	Yes	4 (22)
	No	9 (50)
Treatment	QT	3 (17)
	RxT	1 (5.5)
	QT+RxT.	12 (67)
	No	
Surgery type	Quadrantectomy	6 (33)
	Mastectomy	10 (55.5)
Staging^b^	1	3 (17)
	2	9 (50)
	3	4 (22)
Tumor size^c^	1	4 (22)
	2	5 (28)
	3	7 (39)
Histological grade^d^	1	0
	2	2 (11)
	3	14 (78)
Nuclear grade^e^	1	0
	2	1 (5.5)
	3	15 (83)
CK 5-6 positivity	Yes	10 (55)
	No	‒
	NA	6 (33)
Ki 67	≤ 20%	1 (5.5)
	> 20%	9 (50)
	NA	6 (33)
Inflammatory infiltrate	Yes	7 (39)
	No	8 (44)
Relapse	Yes	4 (22)
	No	12 (67)
Death	Yes	3 (17)
	No	13 (72)

a Data missing for 2 patients.

b 1 = I, 2 = IIA, 3 = IIB+

c 1 ≤ 2 cm, 2 ≥ 2.1–5, 3 ≥ 5 cm

d 1 = I, 2 = II, 2 = III

e 1 = low, 2 = intermediary, 3 = high.

No significant association was observed between patient features and the molecular data for the LA and TN breast cancer samples. We tested the sample size using all the cases for association analyses, but only the Progesterone Receptor (RP) expression and tumor number (multifocal or single nodule) presented significant p values. For RP expression association with *miR-96-5p* and *182-5p*, we found p = 0.002 and p = 0.008, respectively. For the number of nodules, p = 0.036 for *miR-96-5p*.

## Discussion

We detect differential expression in several miRNAs sequences, comparing tumor tissues (LA and TN) with normal breast tissues. Among them, two important FOXO3a negative regulators were identified, *miR-96-5p and 182-5p*. The FOX-O family of proteins is involved in several cellular processes, including tumor suppression, inactivating the proliferation of abnormal cells, repairing DNA damage, and inducing apoptosis. The expression profiles of the specific members of this family vary among different tissues and are regulated in a spatiotemporal manner during the various stages of development.^26^

The FOXO proteins are also involved in the regulation of gene expression in physiological events, such as glucose metabolism, resistance to oxidative stress, and longevity. Among several members of this family, FOXO3a is considered to be a tumor suppressor due to its action in apoptosis, cell cycle arrest, oxidative stress resistance, and autophagy process.^27^ Apparently, FOXO3a represents an adaptable player in the dynamic homeostasis both in the normal tissues and the pathogenic process.^28^^,^^29^ Usually, loss of function of FOXO3a determines the dysregulation in cell proliferation and DNA damage accumulation, resulting in tissue disorders and the development of several types of cancer (including breast and prostate cancer, glioblastoma, rhabdomyosarcoma, and leukemia).^19^^,^^21^^,^^22^

One relevant mechanism involved in the regulation of FOXO3a are the miRNAs that repress the translation of and/or promote the degradation of mRNA.^29^ The 3′-UTR (3′-Untranslated Region) of *FOXO3a* mRNA has several target sequences for miRNAs. FOXO3a can also be regulated by miRNAs in a direct or indirect way.^18^^,^^19^^,^^27^ As mentioned above, our data show the upregulation of *miR-96-5p* in both tumor types, similar to those previously described for breast cancer cell lines.^30^ Lin et al.^31^ also reported increased expression of *miR-96* in breast cancer tissues and cell lines compared to normal breast tissues and epithelial cells. The ectopic expression of *miR-96* induced the proliferation and growth of breast cancer cells, while its inhibition inhibited these effects. This *miR-96* overexpression in breast cancer cells resulted in the modulation of the transition of G1/S cells due to the decreased expression of CDKs (cyclin-dependent kinases) p27 Kip1 and p21 and the increased expression of Cyclin D1. Moreover, *miR-96* inhibited *FOXO3a* expression by binding directly to its 3′UTR.^19^^,^^27^, ^28^, ^29^, ^30^, ^31^

*miR-96-5p* was also overexpressed in tumors and serum from patients with ovarian cancer, which is a relevant hormone-dependent gynecologic cancer.^32^ In colorectal carcinomas, *miR-96* contributed to cell growth, directly targeting *TP53INP1, FOXO1*, and *FOXO3a*.^33^ Taken together, our data and the literature ones, indicate that *miR-96* could play an important role in the proliferation of cancer cells and indicate its potential to be used in miRNA-based therapies for TN patients.

Regarding *miR-182-5p*, our data corroborate the results reported by Krishnan et al.,^34^ who found its upregulation in several human breast cancer samples. The authors identified more than 1,000 target genes for this miRNA, including oncogenes and tumor suppressors, components of the DNA-dependent *BRCA1* repair pathway, and the G2 cell cycle checkpoint genes. In the context of breast cancers, the primary effect of *miR-182-5p* is to mediate the response to DNA damage. Its tumorigenic action was previously described in melanomas^35^ and endometrial cancer.^36^ *miR-182-5p* was also found upregulated in lung, prostate, and colon cancer.^37^ Despite the altered expression of this miRNA in several human cancers, only a few gene targets have been reported until now, including FOXO3.^20^^,^^34^ *miR-182-5p*, along with other miRNAs from the same cluster, showed a relevant effect on apoptosis, senescence, proliferation, and migration/motility in medulloblastoma cells.^38^ Additionally, *miR-96* and *miR-182* coordinate the regulation of genes involved in the apoptotic response, cell cycle checkpoints, and cancer cell metabolism.^30^^,^^31^

We found that in tumors with upregulation of *miR-96* and *miR-182*, there was also a decrease in FOXO3a expression. Taylor et al.^21^ also analyzed the role of the transcription factor FOXO3a in TN breast tumors and concluded that its expression could serve as a better prognostic marker, especially in tumors positive for Estrogen Receptors (ERs).

**Fig. 6:**
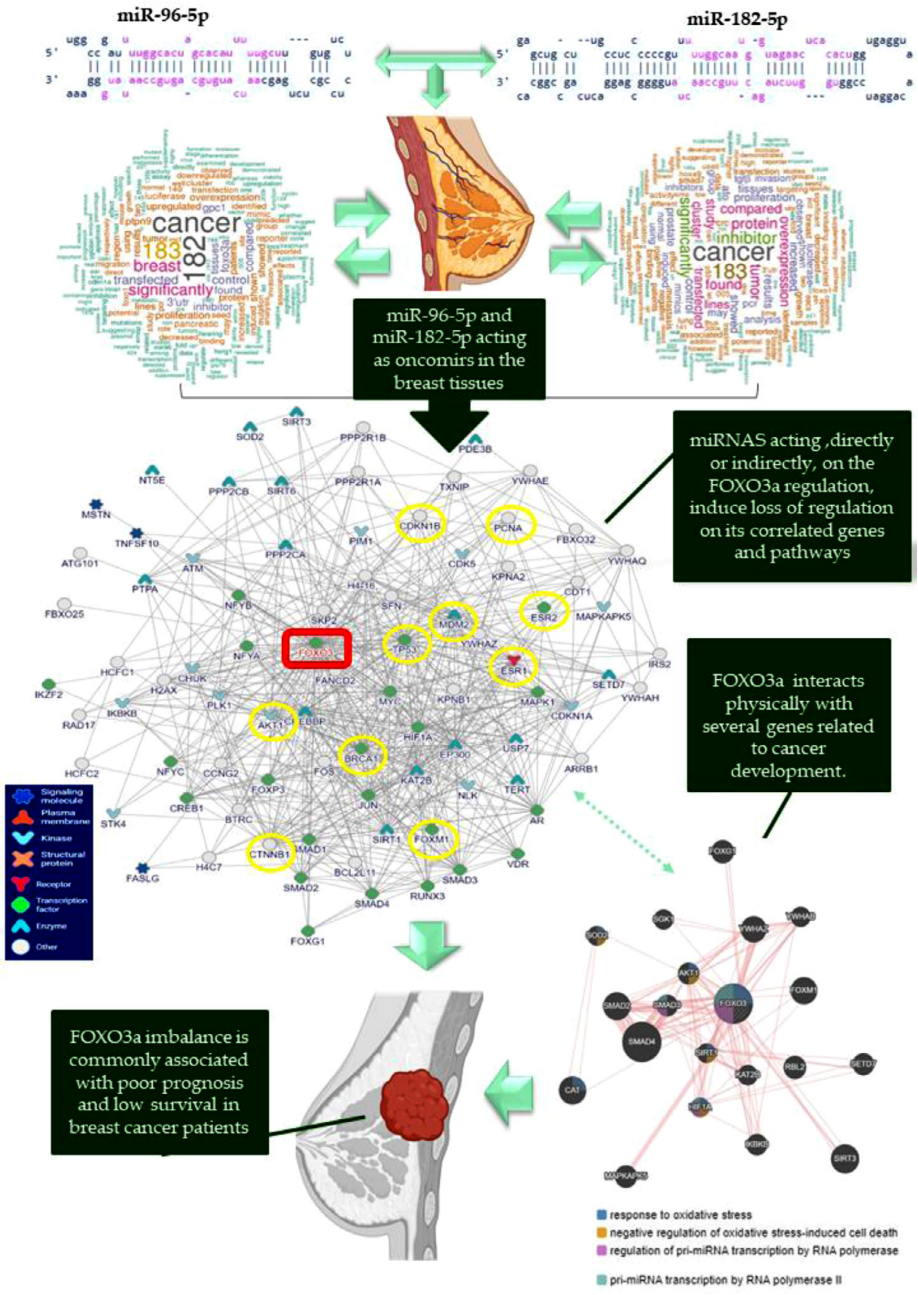
Theoretical representation of *miR-96-5p* and *miR-182-5p* roles as oncomirs in mammary tissues. FOXO3a interaction network is a direct target for several miRNAs, including miR-96-5p and miR-182-5p, resulting in the dysregulation of several key genes. The loss of these intergenic signals due to post-translational regulation by miRNAs is well-characterized in several cancer types, including breast cancers. Several biological processes are compromised when FOXO3a is inhibited, leading to cancer development and patients’ poor prognosis.

As mentioned before, the FOXO3a protein plays a vital role in the regulation of a variety of cellular processes, and its localization in the cell´s compartment is important for its functions. Its phosphorylation leads to its translocation from the nucleus to the cytoplasm, leading to its degradation.^25^, ^26^, ^27^ We did not find the relevant cytoplasmic expression of FOXO3a, and only nuclear expression was identified and quantified. These data support the hypothesis that in these tumors, the upregulation of *miR-96-5p* and *miR-182-5p* are responsible for the inhibition of the FOXO3a signaling and several oncogenic pathways activation (Fig. 6).

The multiple functions of FOXO3a indicate that its dysregulation is associated with several diseases, including cancer. Its upregulation has already been associated with the inhibition of tumorigenesis in breast cancer.^39^^,^^40^ Therefore, exploring the network of interactions between miRNAs and FOXO3a may help find strategies to support the future of breast cancer.

Despite of several studies focusing in FOXO3a role in breast cancer, sometimes as a tumor suppressor other times as a poor prognostic marker, no evidences about its role is avaible for Brazilian women. Here, we found that, FOXO3a expression in our sample set corroborate the literature with FOXO3a presenting a suppressor profile.

To the best of our knowledge, this study is the first evidence of the interacting role of FOXO3a and miR-96/miR-182. Our results showed the importance of these molecules in breast cancer, especially in TN tumors, even with the limitations due to the technique and samples used. Our main difficulties were extracting genetic material with sufficient quality (integrity) and quantity (concentration) from the paraffin-embedded tissues. There was a loss in the number of cases due to RNA degradation and also due to the final amount of the obtained RNA. In the Real-Time PCR reactions, there was also a reduction in the samples ´number, due to the controls included in the array that filter the results based in the genomic DNA contamination, PCR and cDNA synthesis efficiency and housekeeping genes amplification. However, these controls made the results more reliable and reproducible, as can be seen in a very recent work of Kandil et al., (2022). The authors assessed FOXO3a (RNA) and *miR-182* expression in 25 samples of invasive ductal carcinoma (IDC) presenting different histological grades (GI to GIII). Their concluded that FOXO3 down regulation and *miR-182* upregulation are associated with advanced breast cancer. These results corroborate and reinforce our results.^41^

## Conclusions

Our data show that in triple negative breast cancer with high expressions of *miR-96-5p* and *miR-182-5p*, the FOXO3a protein is inhibited. Further studies including a large number of samples are necessary to establish the role of these molecules in Brazilian´s breast cancer women.

## Authors' contributions

Daniele Carvalho Calvano Mendes: Performed the experiments, data analyses and manuscript preparation.

Carlos Marino Cabral Calvano Filho: Performed the experiments, data analyses and manuscript preparation.

Natália Garcia: Performed IHC reactions and data analyses.

Marcos Desidério Ricci: Original project idea conception and intellectual support.

José Maria Soares Júnior: Manuscript revision and intellectual support.

Katia Candido Carvalho: Data analysis, intellectual support and manuscript submission.

Edmund Chada Baracat: Original project idea conception, intellectual support and manuscript revision.

DCCM, CMCCF, and KCC designed the study, performed the experiments, analyzed the data, and wrote the manuscript. NG performed IHC reactions and data analyses. MDR and ECB de-signed the study and intellectual support.

## Funding

The study was supported by Fundação de Amparo à Pesquisa do Estado de São Paulo (FAPESP nº 2010/16824-4).

## Institutional review board statement

The study was conducted in accordance with the Declaration of Helsinki, and approved by the Institutional Review Board – CAPPesq of the Hospital das Clinicas da Universidade de São Paulo (Protocol number: 0042/11).

## Informed consent statement

Informed consent was obtained from all alive subjects involved in the study.

## Data availability statement

Not applicable.

## Declaration of Competing Interest

The authors declare no conflicts of interest.
